# Measurement of cardiovascular function using a novel view-sharing PET reconstruction method and tracer kinetic analysis

**DOI:** 10.1186/s40658-016-0161-4

**Published:** 2016-10-20

**Authors:** Paul R. Territo, Amanda A. Riley, Brian P. McCarthy, Gary D. Hutchins

**Affiliations:** Department of Radiology and Imaging Sciences, Indiana University School of Medicine, 950 W. Walnut St, R2 E124, Indianapolis, IN 46202 USA

**Keywords:** Cardiac output, PET, View-sharing, Kinetic modeling

## Abstract

Recent advancements in PET instrumentation have made the non-invasive assessment of cardiovascular function in small animals a reality. The majority of small animal PET systems use stationary detector gantries, thus affording high temporal resolution imaging of cardiac function. Systems designed to maximize spatial resolution and detection sensitivity employing rotating gantry designs are suboptimal when high temporal resolution imaging is needed. To overcome this limitation, the current work developed a novel view-sharing data analysis scheme suitable for dynamic cardiac PET imaging using ^18^F-NaF as the tracer and tracer kinetic model analysis. This scheme was tested in a rat model of cardiovascular function where the relationship between direct transonic flow measures of cardiac output were highly correlated (*f*(*x*) = 1.0216*x* − 24.233, *R* = 0.9158, *p* < 0.001) with the new model. Similarly, derived measures of stroke volume were also highly correlated (*f*(*x*) = 0.9655*x* − 0.0428, *R* = 0.9453, *p* < 0.001) with the current approach. Administration of xylazine caused a statistically significant increase in stroke volume (0.32 ± 0.07 ml, *p* = 0.003, *n* = 4) and a significant decrease in both heart rate (−155 ± 7.1 beats/min, *p* < 0.001, *n* = 4) and cardiac output (−75.9 ± 23.0 ml/kg min, *p* = 0.01, *n* = 4). These findings suggest that the new sinogram binning and kinetic modeling methods produce reliable cardiac function measures suitable for longitudinal monitoring of cardiovascular function.

## Introduction

Determination of cardiovascular function in small animals has been well characterized using a number of in vivo model systems. In particular, the application of the direct Fick principle [1], employing electromagnetic flow meters [2], indicator dilution [3], and thermal [4] dilution methods have been used with great success. The invasive nature of these techniques makes them better suited for acute rather than longitudinal measurements of function. To overcome this limitation, non-invasive small animal quantitative PET methods have been developed for both rats [5–10] and mice [11]. Primary challenges in quantifying cardiac function via small animal PET include limited spatial resolution and suboptimal detection sensitivity of these devices. Currently, a majority of the small animal PET scanners in use have a fixed ring geometry and yield a spatial resolution at the center of the field of view (CFOV) in the range of 1.3–1.7 mm full width at half-maximum (FWHM) and a overall system sensitivity of 3–5 % [7, 12–14]. Kreissl et al. [11] acquired cardiac PET data in listmode and reconstructed the images into short-duration frames (0.3 s/frame) in order to measure the first pass kinetics of the radiotracer through the heart. In order to gain greater spatial resolution and system sensitivity, small animal PET scanners, like IndyPET3, have been developed with a slip-ring geometry which encodes all events spatially as the gantry rotates in order to increase spatial sampling by using 12 coincidence banks, thus yielding resolution at CFOV of <1 mm while simultaneously maximizing sensitivity (>7 %) with long detector crystal lengths [15]. Although these advances provide substantial benefit for cardiac PET imaging, the use of high temporal resolution frame rates [11] with a rotating gantry configuration will eliminate the resolution gain provided by the unique spatial sampling schemes employed in these systems. The objective of the current study was to develop a novel sinogram binning and data processing scheme that enables rapid cardiac function studies to be performed while maintaining the improved spatial resolution produced by small animal PET systems that incorporate a rotating gantry. To test this approach, we developed an animal model that enabled simultaneous measurement of cardiac function with a transonic flow meter and PET determination of function using tracer kinetic models which utilizes the sinogram binning.

## Materials and methods

### Experimental design

**Fig. 1:**
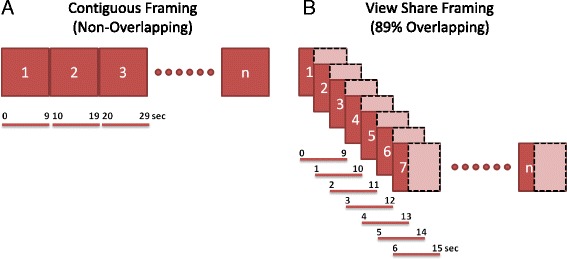
Visual representations of ultra-short contiguous (non-overlapping) frame reconstruction [11] (**a**) and view-sharing (89 % overlapping) frame reconstruction (**b**) used with stationary and rotating gantry PET systems, respectively

All procedures were approved by the Indiana University School of Medicine Animal Care and Use Committee prior to the start of the study and were conducted in accordance with NIH guidelines on animal care and use [16]. Four adult Sprague-Dawley male rats were anesthetized using isoflurane (3–4 % isoflurane), balanced with medial grade oxygen at a flow rate of 2 l/h. Once anesthetized, animals were placed on custom carbon fiber bed and maintained at 1.5–2 % isoflurane during surgical preparation of the abdomen. A small incision was made over the midline, the skin retracted, and abdominal contents displaced laterally and sterile saline soaked gauze was placed over contents to ensure hydration. Using blunt dissection, the abdominal aorta was isolated from the surrounding nerve and vena cava, and a transonic flow probe (TFP;Transonic, Model # PRB3313) was placed as described previously [2, 17]. Abdominal contents were replaced into the cavity and the skin was closed. Animals were quickly transported to the imaging suite and positioned for the imaging studies (imaging bed mounted, flow probe, and ECG leads connected). Isoflurane (1–3 % isoflurane) anesthesia was maintained for the duration of the imaging session. Venous access was secured via tail vein catheterization, and baseline readings of cardiac output and heart rate were collected in triplicate. Immediately after starting PET data acquisition, animals were delivered a dose of ^18^F-NaF (14.8 ± 0.48 MBq/kg, *n* = 4) IV, followed by repeat collection of heart rate and cardiac output measurements in triplicate. ^18^F-NaF was selected due to its rapid vascular clearance (facilitated by hydroxyapatite binding in the bone), minimizing the cross-talk between the repeat ^18^F-NaF dose administrations. In all cases, dynamic PET images were acquired in listmode using the IndyPET3 scanner [18] with a gantry rotation rate of 3 rpm. With this configuration, a minimum rotation angle of 60°, which requires minimum scan duration (MSD) of 3.33 s, is required for acquisition of a uniformly sampled data set suitable for sorting into a high-resolution sinogram and application of analytical image reconstruction algorithms. Following the baseline scan, cardiac output was manipulated by administering the α_2_-agonist xylazine via constant rate infusion at 13.8 mg/kg min [19]. Once at steady state, animals were administered a second dose of ^18^F-NaF (15.5 ± 0.1.7 MBq/kg, *n* = 4) IV, followed by repeat collection of cardiac output and heart rate in triplicate. Dynamic listmode data (15 min/treatment) were reconstructed into 80 × 100 mm volumes using filtered back projection (FBP). In order to maintain the high intrinsic spatial resolution of the IndyPET3 system, with signal-to-noise ratios (SNR) yielding acceptable image quality, while capturing rapid tracer kinetic changes in the cardiac LV chamber, listmode data were binned using a temporal overlap design with 9 s (3*MSD) duration frames offset by 1 s. Using this schema, frames 1, 2, 3, through “*n*” would yield the following [start, stop] frame integration times: [0, 8], [1, 9], [2, 10], and [*n*, *n* + 8], respectively. The result is a 4D series with 89 % view-sharing per frame (see Fig. 1). In all cases, images were corrected for decay, random coincidence events, and dead-time loss [20]. Animal beds were transferred to the EVS R9 microCT, and images were acquired center over the heart using 80 kVp, 1000 mA, 200 ms/°, 1.08°/step, and reconstructed into an 40 × 65 mm volumes using vendor-supplied FBP reconstruction algorithm.

### Image analysis

**Fig. 2:**
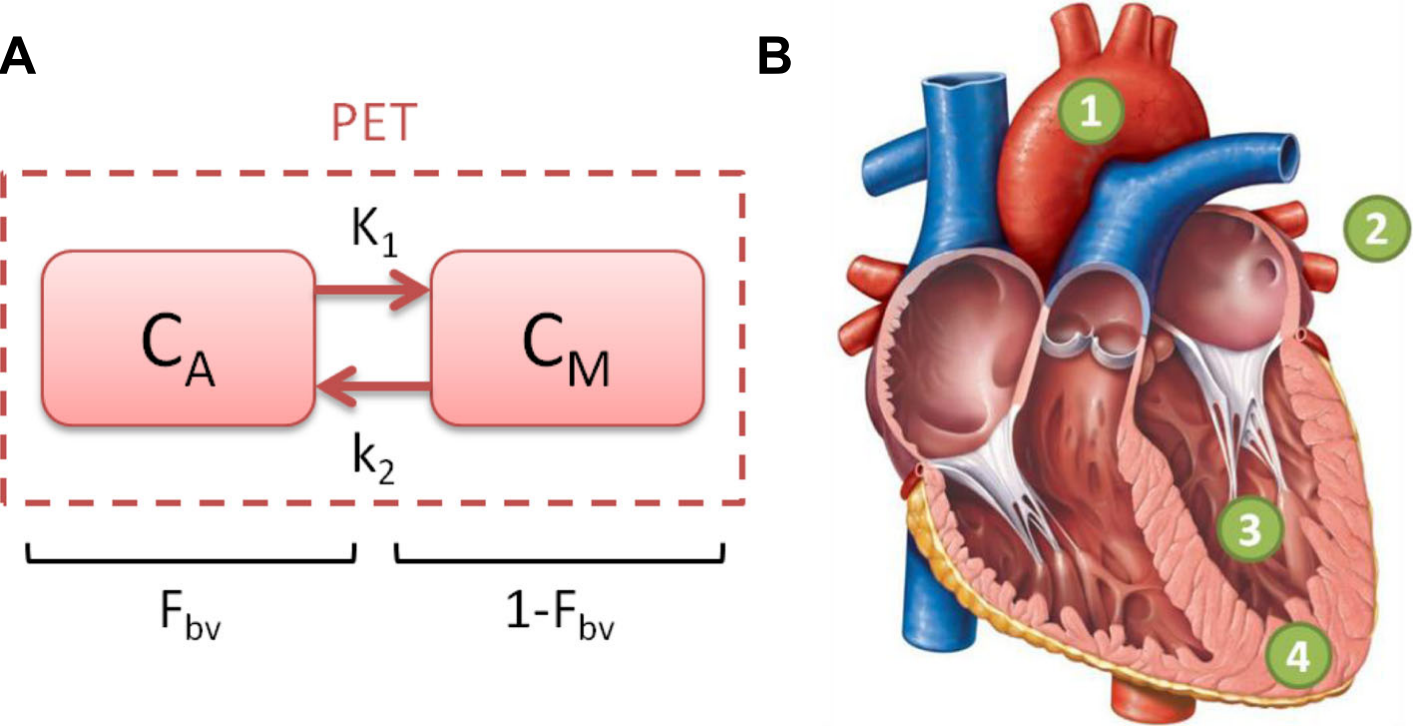
**a** Schematic diagram of the *1* compartment *3* parameter tracer kinetic model. **b** Heart diagram indicating the points of reference as described in Eqns. 1–6 (reproduced with permission from Pearson Education Inc.)

PET and CT images were imported, reoriented, and registered using a normalized entropy algorithm [21]. Using registered CT images, the left ventricular cavities were manually segmented avoiding the apex, septum, and free walls to limit the ventricular spillover. Regions of interest were then extracted from the PET time series, and a tracer kinetic model describing ^18^F-NaF kinetics in LV blood and myocardial tissue (Fig. 2a) was fit to the data. The ^18^F-NaF concentration measured in the LV VOI was described by the following equation.

1 $$ {C}_{\mathrm{PET}}\left(t(i)\right)=\frac{1}{\left({t}_2(i)-{t}_1(i)\right)}\left(1-{F}_{\mathrm{bv}}\right){\displaystyle {\int}_{t_1(i)}^{t_2(i)}{C}_M\left(\tau \right)d\tau +{F}_{\mathrm{bv}}}{\displaystyle {\int}_{t_1(i)}^{t_2(i)}{C}_A\left(\tau \right)d\tau } $$

where *C*_PET_(t(*i*)) is the measured ^18^F-NaF concentration in the LV VOI, *C*_*M*_(*t*(*i*)) is the myocardial tissue ^18^F-NaF concentration (Fig. 2b. marker﻿ 4, Eq. 5), *C*_*A*_(*t*(*i*)) is the arterial blood ^18^F-NaF concentration (Fig. 2b. marker 1, Eqs. 2, 3, and 4), *F*_bv_, is the fractional blood volume, and “*i*” represents the temporal frame number, respectively. The pulmonary artery *C*_PA_(*t*) (Fig. 2b. marker 2, Eq. 3) input to the LV and the LV response function (LV_rf_(*t*)) (Fig. 2b. marker 3, Eq. 4) determine the arterial blood concentration (Eq. 2)

2 $$ {C}_A(t)={C}_{\mathrm{PA}}(t)\otimes {\mathrm{LV}}_{\mathrm{rf}}(t) $$

3 $$ {C}_{\mathrm{PA}}(t)=Ae\frac{-{\left(t-{t}_0\right)}^2}{2{\sigma}^2} $$

4 $$ {\mathrm{LV}}_{\mathrm{rf}}(t)=\frac{\overset{.}{Q}}{{\mathrm{V}}_{\mathrm{lv}}}{e}^{-\left(\frac{\overset{.}{Q}}{{\mathrm{V}}_{\mathrm{lv}}}\right)t} $$

5 $$ {C}_M(t)={K}_1{C}_A(t)\otimes {e}^{-{k}_2t} $$

6 $$ \overset{.}{Q}=\mathrm{S}\mathrm{V}{f}_h $$

where *A*, *t*_0_, *σ*, $\overset{.}{Q}$, SV, f_h_, V_lv_, K_1_, and k_2_ are the input amplitude, time offset, input width, total cardiac output (ml/g min), stroke volume (ml), heart rate (beats/min), left ventricular volume (ml), tissue perfusion times extraction fraction (ml/g min), and back flux rate constant (1/min), respectively. For Eqs. 2 and 5, ⊗ represents the convolution operator. As indicated in Eq. 1, each image frame (*i*) is constructed by integrating over the interval [*t*_1_(*i*), *t*_2_(*i*)]. For this study, *t*_2_(*i*) = *t*_1_(*i*) + 8.

### Statistics

Statistical analysis was performed on direct measures of TFP and tracer kinetic model estimates of stroke volume (SV) and cardiac output (CO) parameters, where heart rates (HR) were collected simultaneously for both measures. Correlation analysis was performed on all measures of SV and CO using Pearson-product moment correlation analysis. Statistical analysis was performed as two-tailed paired *T* test pre- and post xylazine in fusion, where significance (*) was taken at *p ≤* 0.05.

## Results

**Fig. 3:**
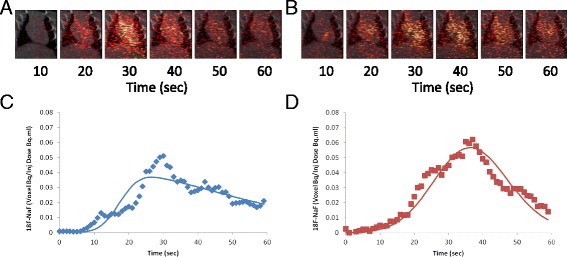
Fused ^18^F-NaF PET/CT view-sharing reconstructed images with time of the heart prior to (**a**) and post (**b**)-xylazine infusion at 13.8 mg/kg min, where the corresponding kinetic time courses pre (**c**) and post (**d**) are shown below each image series, where *solid line* represent the fit of the described model. Reconstructions utilized a 9-s frame duration with a 1 s offset as described in Fig. 1

**Fig. 4:**
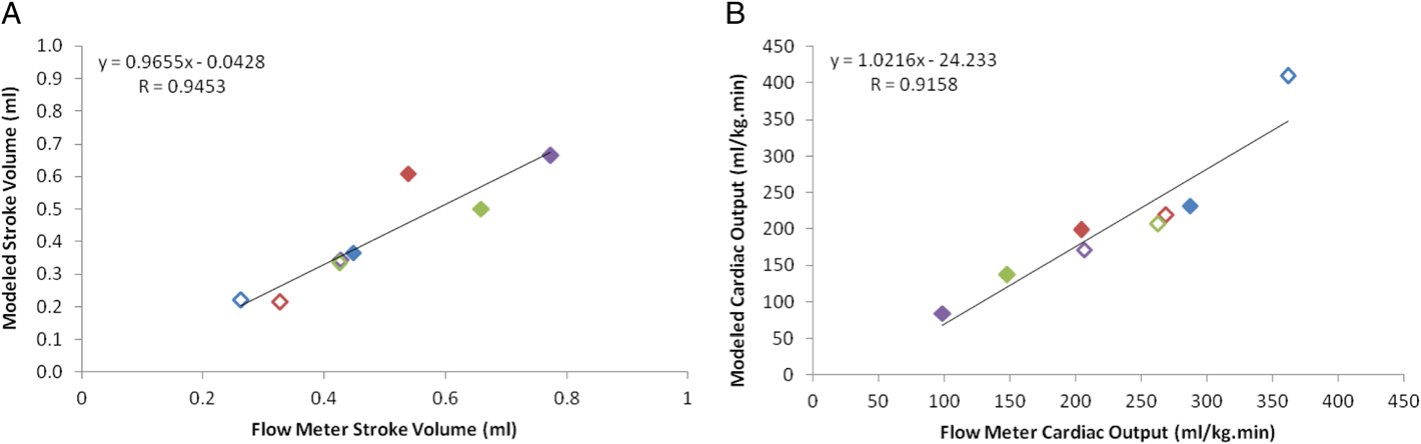
Comparison of flow meter and tracer kinetically modeled **a** stroke volume and **b** cardiac output at baseline and postxylazine infusion at 13.8 mg/kg min [19]. The equation in each chart represents the linear relationship between the measures, while the measure of fit is provided by the Pearson product-moment correlation coefficient (*R*). In all cases, *unique colors* represent individual animals at baseline (*open symbols*) and post xylazine (*filled symbols*) treatment

Images acquired over the first 60-s post ^18^F-NaF administration and reconstructed using the 89 % temporal view-sharing scheme, as described in Fig. 1, are shown in Fig. 3a-b. Segmentation of the PET/CT images produced dose normalized TACs consistent with the baseline and xylazine treatments (see Fig. 3c, d). These time courses when fit with the kinetic model described by Eqs. 1–6 yielded estimates of the left ventricular SV and total CO at baseline and following xylazine administration. To determine the relationship between the TFP measures and PET kinetically derived parameters, correlation analysis was performed for SV (Fig. 4a) and CO (Fig. 4b). The relationship between TFM measurements and PET estimated SV can be described by *f*(*x*) = 0.9655x − 0.0428, with a correlation coefficient of *R* = 0.9453 (*p* < 0.001, *n* = 4, groups = 2). Similarly, the correlation coefficient between TFM measurements and PET estimates was *R* = 0.9158 (*p* < 0.001, *n* = 4, groups = 2) and can be described by *f*(*x*) = 1.0216x − 24.233. When analyzed by treatment, SV significantly increased from 0.30 ± 0.05 ml to 0.62 ± 0.06 ml (*p* = 0.003, *n* = 4, groups = 2) when measured via TFM, while PET estimates yielded a similar increase from 0.30 ± 0.04 ml to 0.54 ± 0.04 ml (*p* = 0.003, *n* = 4, groups = 2). As expected, the infusion of xylazine resulted in a significant decrease in CO by (75.9 ± 23.0 ml/kg min (*p* = 0.017, *n* = 4, groups = 2) as measured by TFM, with PET showing a similar reduction over this same interval 84.1 ± 18.1 ml/kg min (*p* = 0.007, *n* = 4, groups = 2). Concomitantly, HR over this same interval decreased by 155 ± 7.1 beats/min (*p* < 0.001, *n* = 4, groups = 2)

## Discussion

Over the past decade, there is an emerging interest in dynamic cardiac PET as a tool to assess small animal cardiovascular function [5–11]. Spatial and temporal resolution presents a key challenge for quantifying cardiac function in rodents. Currently, the majority of the small animal PET scanners rely on a fixed ring geometry which yields a spatial resolution at CFOV of 1.3–1.7 mm FWHM and an overall system sensitivity of 3–5 % [7, 12–14]. Taking advantage of this fixed geometry, Kreissl et al. [11] acquired cardiac PET data in listmode and reconstructed these into short frame durations (0.3 s/frame), thereby permitting quantification of the first pass of the radiotracer through the heart. An important consideration of fixed ring geometry systems is that they cannot achieve the inherent spatial resolution of the PET detectors when employing traditional FBP algorithms due to limited spatial sampling constraints. Advancement in scanner design which employ a slip-ring geometry along with spatial encoding of events as the gantry rotates, resulting in resolution and sensitivity at CFOV of <1 mm and >7 % [15], respectively. These advances provide substantial benefit for small animal cardiac PET imaging; however, the use of high temporal resolution PET techniques as previously described [11] (i.e., 0.3 s/frame) will sacrifice the spatial resolution gain achieved through the rotating gantry design. Therefore, the current study builds upon the prior advancements in instrumentation and reconstruction [11] approaches and develops a novel view-sharing sinogram binning scheme that provides high temporal sampling of images for rotating gantry PET systems and while maintaining the spatial resolution advantages of a rotating gantry design.

This novel sinogram binning approach when used with listmode acquisitions provides high temporal resolution by overlapping the image frames, where the image projection content is a fixed proportion from prior and newly acquired projection data (Fig. 1b). Although this overlapping scheme permits high temporal resolution while maintaining high spatial resolution, the resulting image frames can be described as the convolution of the image series with a boxcar function specified by the overlap length. This resulting tissue VOIs curves are fit with a tracer kinetic model (Eqs. 1, 2, 3, 4, and 6) using integration limits [*t*_1_(*i*), *t*_2_(*i*)] that are consistent with the data binning process. To test this reconstruction and analysis approach, we developed an animal model that enabled PET-based cardiac function estimates to be validated against a transonic flow meter. Estimates of SV and CO prior to and postxylazine infusion could be reliably estimated using the view-sharing PET methodology, as demonstrated by the strong correlation with the TFP measurements (Table 1). Our results were consistent with prior literature reports using the direct Fick method [1], thermal/indicator dilution [3, 4], and TFM [2] measures.

**Table 1:** Tracer kinetic modeling statistics prior to and post xylazine. Data are presented as mean ± SEM

Group	K1 (ml/g.min)	SEM	k2 (1/min)	SEM	*n*
Baseline	3.67	1.16	0.975	0.597	4
Xylazine	6.45	2.84	1.431	0.576	4

These data show for the first time that high temporal sampling of PET images, view-sharing sinogram binning scheme, and tracer kinetic analysis of PET series are feasible for monitoring dynamic cardiac functional changes in a rotating gantry PET scanner. Moreover, we demonstrated that over a broad range in SV and CO changes, the current method provides a rapid assessment of the heart function not previously possible with rotating gantry PET scanners. When coupled to a scanner with high sensitivity and spatial resolution [15], this approach provides a key opportunity to leverage the system features along with high temporal resolution imaging for assessment of cardiovascular function in small animals.
